# Treatment Equality May Lead to Harmonious Patient–Doctor Relationship During COVID-19 in Mobile Cabin Hospitals

**DOI:** 10.3389/fpubh.2021.557646

**Published:** 2021-06-15

**Authors:** Zhi-Peng Xu, Jun-Jian Zhang, Nao Yan, Hu Yingying

**Affiliations:** Department of Neurology, Zhongnan Hospital of Wuhan University, Wuhan, China

**Keywords:** enlightenment, relationship, COVID-19, hospital, China

The doctor–patient relationship is crucial to the healthcare delivery. In recent years, the growing problem of violence against doctors in China suggested that the doctor–patient relationship had become increasingly tense and complicated (1–3). China's first fundamental and comprehensive law on basic medical and healthcare have taken effect on June 1, 2020, including protecting the safety of medical personnel (4). During the coronavirus disease 2019 (COVID-19) outbreaks, Wuhan, the epicenter of the virus, built 16 mobile cabin hospitals (each holds 500–2,000 beds) and created more than 10,000 beds for mild cases to ease the ongoing shortage of the city's treatment resources. A cabin hospital is a mobile medical structure easily shipped and installed and widely used with multiple functions, such as emergency treatment, surgical treatment, and clinical examination. Remarkably, patients and doctors in mobile cabin hospitals got along better and were able to build a relaxed and friendly relationship with each other. Undoubtedly, this phenomenon is not accidental at all and brings better understanding on how to relieve the tensions of relationships between doctors and patients.

Mobile cabin hospitals are a realistic way to solve the core shortcoming of centralized admission and treatment during COVID-19. Mobile cabin hospitals are not only medical facilities but also the “cozy homes” for patients. Patients and doctors in mobile cabin hospitals maintain a friendly relationship. There are several reasons for this.

First, mobile cabin hospitals are real non-profit public hospitals providing free medical care. The cabin hospital is equipped with lab tests and X-ray machines, which can monitor the condition of patients without charge. The patients no longer need to worry about their payment for medical services, overexamination, or unnecessary treatments. In recent years, with the rapid development of market economy, some public hospitals have expanded the services they provide, purchased large medical equipment, and blindly pursued economic benefits, which embarrassingly made medical treatment more difficult and expensive (5). Public hospitals should focus on the health and care of their patients rather than hospitals' financial performance. Hospitals should return back to their actual tasks of patient care and establishing a good patient–doctor relationship.

Second, mobile cabins provide equal treatment for all patients in need. Every mobile cabin hospital is like a Noah's Ark that cares for large numbers of COVID-19 patients with mild symptoms in a short time period. During the time of their treatment, patient in care of mobile cabins participate in the decisions about their treatment and help each other. At present, hospitals in cities have many staff and technical resources, whereas hospitals in rural or remote areas are short of medical personnel and basic medical equipment. The uneven distribution of medical resources has caused a great deal of tension and seriously affected the relationship between doctors and patients.

Third, equal participation and no barriers to access to treatment in mobile cabin hospitals are beneficial for the doctor–patient relationship. The doctors share responsibility for making decisions and planning the course of treatment in mobile cabin hospitals. The patients actively share information, their feelings and thoughts, and are willing to accept their health team's instructions. Besides antivirus therapy, more than 85% of patients have considered traditional Chinese medicine (TCM) as another treatment for COVID-19. Some patients engage in activities led by doctors that are equally satisfying to both parties, such as playing Tai Chi, dancing like the Uygurs, or presenting self-made short films. Some patients sit in the reading corner and enjoy reading newspaper or magazine. Previous studies have revealed that patient engagement and cooperation led to improvements in patient satisfaction and outcomes (6).

Fourth, communication without barriers is like a bridge of understanding between doctors and patients in mobile cabin hospitals. All patients have been newly diagnosed with COVID-19 infection, and they know that there is currently no specific treatment approved for COVID-19. The doctors communicate with the patients to ensure their rights to be informed and encourage them to be active, instead of being fearful. The patients report their symptoms to the doctors to modify their treatment plan. When patients seem to be overly stressed, the doctors provide psychological counseling and treatment for the patients in mobile cabin hospital. Cooperation and mutual respect help doctors and patients to communicate and cooperate well. The improvement of doctor–patient communication can strengthen the doctor–patient relationship and the quality of healthcare (7).

Fifth, positive media reports affect the perceptions of the doctor–patient relationship in mobile cabin hospitals. Social media has an even stronger impact on doctor–patient relationships. According to an analysis with propensity-score matching (8), national media reporting of “bad news” seems to negatively affect the perception of doctor–patient relationships among both patients and doctors in China. Strategies to improve the doctor–patient relationship might need to include media management. Nevertheless, national media reporting of good news brought them hope and encouragement and a sense of calmness, which instantly enhanced their mood in mobile cabin hospitals. The doctors and patients were extremely grateful for this positive outcome, which is also beneficial for improving public perceptions of the doctor–patient relationship.

In brief, patient dissatisfaction is derived from expensive fees, disparity of healthcare resources, lack of engagement and cooperation, poor communication, and impact of adverse media reporting. Therefore, it is vital to speed up the development of medical insurance, to realize that all people are equal in their rights to receive medical treatment and need equal rights to access healthcare without barriers, to enhance mutual understanding and cooperation, strengthen the implementation of effective and informative communication, and promote positive media reporting on medical service in Chinese public hospitals.

Equal rights in provision and treatment may have led to harmonious patient–doctor relationship during COVID-19 in mobile cabin hospitals. With proper measures in place, a harmonious and pleasant doctor–patient relationship is likely to emerge in all public hospitals.

## Author Contributions

Z-PX involved in design, drafting, and revising the manuscript. J-JZ involved in design and revising the manuscript. NY and HY contributed to manuscript revision.

## Conflict of Interest

The authors declare that the research was conducted in the absence of any commercial or financial relationships that could be construed as a potential conflict of interest.
